# Atropa Belladonna intoxication: a case report

**Published:** 2012-04-17

**Authors:** Mohamed Adnane Berdai, Smael Labib, Khadija Chetouani, Mustapha Harandou

**Affiliations:** 1 Intensive Care Unit, Mother and Child Hospital, University Hospital Hassan II, Fes, Morrocco

**Keywords:** Intoxication, atropa belladonna, child, anticholinergic toxidrome, atropine

## Abstract

*Atropa Belladonna* is a poisonous plant also called deadly nightshade. Its roots, leaves and fruits contain alkaloids: atropine, hyocyamine and scopolamine. The risk of poisoning in children is important because of possible confusion with other berries. *Atropa Belladonna* acute intoxication is a severe condition, it's should be considered in the presence of anti-cholinergic toxidrome, the differential diagnosis include other plants or psychoactive drugs containing atropine. The treatment is mainly symptomatic including gastrointestinal decontamination with activated charcoal. In severe cases, physostigmine can be used as an antidote. We report the case of 11 year old girl with *Atropa Belladonna* poisoning which was administrated in a therapeutic purpose as a remedy to jaundice. The child presented essentially a central anti-cholinergic syndrome. She was admitted in the intensive care unit, the progression was favorable with symptomatic treatment.

## Introduction

*Atropa Belladona* is a poisonous plant called deadly nightshade. It's a plant classified in the solanaceae family and its roots, leaves and fruits contain the belladonna alkaloids: atropine, hyocyamine, and scopolamine [1], responsible for the anticholinergic toxicity of the plant. We report an uncommon case of intoxication with *Atropa Belladona* in a child.

## Patient and observation

It's an eleven year old girl, under Rifampicin and Isoniazid for lymph node tuberculosis, who developed jaundice as side effect of this treatment. She was given *Atropa Belladona* by an herbalist in a therapeutic interest. Since then, the patient presented: dry mouth, confusion, incoherent speech, inability to recognize members of the family, she also presented uncontrollable vomiting, visual disturbances, hearing and visual hallucinations. The clinical examination revealed disturbance of consciousness, the coma Glasgow scale (GCS) was evaluated at 13/15, the pupils were equal and reactive, she presented polypnea at 26 cycle per minute, but she was afebrile and hemodynamically stable, with mucocutaneous jaundice, tendon reflexes were sharp and diffuse. Routine full blood count, renal and liver function tests revealed hepatic cytolysis. Chest radiography and electrocardiography were unremarkable. The ingestion time of the poisonous fruits was the previous day so that activated charcoal was not administered. The patient was monitored in a critical care unit for vital findings and Diazepam 5 mg was administrated twice for sedation. She received a symptomatic treatment based on oxygenotherapy, antiemetic, stomach protection and hydro electrolytic supply. The anti tuberculosis treatments was stopped because of its hepatic side effect. The evolution was marked by neurological improvement (GCS:15), disappearance of delirium, regression of jaundice and normalization of laboratory tests.

## Discussion

**Figure 1:**
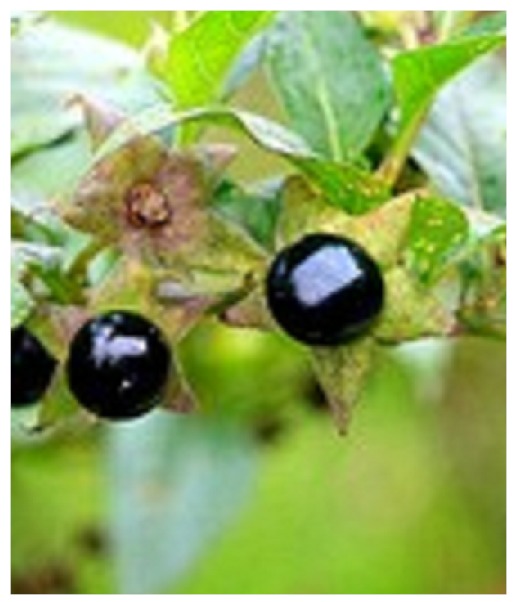
Berries of Atropa belladonna

*Atropa Belladona* is a perennial bushy herb taxonomically classified in the family *solanaceae*, it can grow up to five feet tall and is usually found in quarries and waste ground. The flowers are greenish-purple and the leaves are oval. The berries are black, globular, sweet (Figure 1) and are consumed by animals that disperse the seeds in their droppings [2, 3]. It's a rare plant and dangerous: the ingestion of 10 bays would be toxic to an adult, 2-3 for a child. The risk of poisoning in children is important because of possible confusion with other berries (blackcurrant, blueberry) [4].

The name belladonna comes from the Italian, meaning beautiful lady, originating either from its use as a facial cosmetic, or, more probably, from its use to increase pupil size in women [3]. Since antiquity, the lethal, as well as the hallucinogenic effects of poisoning with *Atropa Belladona* are well known; therefore, they are an important part of orgies and rituals [1]. All parts of the plant contain alkaloids (atropine, hyocynamine and scopolamine), but the highest content is in the ripe fruit and the green leaves. These chemicals act by competitively blocking the binding of acetylcholine to the central nervous system and parasympathetic postganglionic muscarinic receptors [1, 2].

Accidental ingestion of deadly nightshade berries can induce an anti-cholinergic toxidrome. However, not all the characteristics of anti-cholinergic toxidrome may be present in some cases of poisoning due to some plants having a hybrid form. All anticholinergic toxidrome findings may be encountered in *Atropa Belladona* poisoning. Clinical manifestations are caused by central nervous system effects, peripheral nervous system effects, or both [3]. The anticholinergic syndrome is a constellation of signs and symptoms that may be present in whole or in part in the poisoned patient. Central effects are dose-dependent and agent-dependent [5], Patients with central anti-cholinergic syndrome may present with ataxia, disorientation, short-term memory loss, confusion, hallucinations, psychosis, agitated delirium, seizures, coma, respiratory failure or cardiovascular collapse. Patients may present to the emergency department with a psychotic picture [3]. The peripheral effects that are common to anticholinergic agents include mydriasis with cycloplegia, dry mucous membranes, hyperreflexia, flushed skin, diminished bowel sounds or ileus, urinary retention, tachycardia, and hypertension or hypotension [5]. In childhood, meaningless speech, lethargy, coma and absence of tachycardia are the ominous signs in deadly nightshade intoxication [1]. When the history of ingestion of berries is clear, and the plant is rapidly identified there are usually few problems with the diagnosis. However, where the poison has passed through an intermediate animal, then the diagnosis can be difficult and confusing, like in the case of meat from cattle and rabbits which have grazed on *Atropa Belladona* [6]. Cases of deadly nightshade intoxication may also be confused with post-traumatic brain damage, and acute psychosis. Therefore, in case of excitability and confusion, as well as somnolence or coma of uncertain etiology, an anticholinergic syndrome caused by ingestion of atropine-containing plants or psychoactive drugs should be included in the differential diagnosis [1]. Difficulty in diagnosis may arise when the patient has been exposed to a drug with anticholinergic properties like: Antihistamines, tricyclic antidepressants, phenothiazines, antipsychotics, neuroleptics, cyclobenzaprine, antiparkinson drugs, cycloplegics and antispasmodics. Differential diagnosis can also be made with numerous plants with anticholinergic properties like: Jimsom weed (*Datura Stramonium*), Salvia divinorum, Angel's trumpet (*Datura sauveolens*) and Black Henbane (*Hyoscyamus Niger*) [5].

Management is conservative and consists of observation and nursing the patient in a darkened, quiet environment. Activated charcoal adsorbs the agents quite well. Benzodiazepines may be used for sedation if the patient is very agitated [2]. Physostigmine may be useful in severe cases [3], it's a reversible cholinesterase inhibitor, it cross the blood-brain barrier and act on both central and peripheral anticholinergic symptoms [4]. The toxicity associated with physostigmine consists mostly of seizures and cardiac arrhythmia, and occurs when used in the absence of anticholinergic symptoms. Despite potential complications, physostigmine can be beneficial in pure anticholinergic overdose with severe symptoms; it can also be useful by controlling agitation and reversing delirium. The potential for side effects is not insignificant, and it should be used with caution in any patient with unknown ingestions or those with cardiac conduction defects [7]. Some authors propose, in case of no availability of physostigmine, the use of neostigmine even though, it does not pass the blood brain barrier [4].

The particularity of our case, is the association between hepatic side effects of the anti tuberculosis treatment and the manifestations of poisoning by *Atropa Belladona*. Other particularity, the presence of an incomplete atropinic syndrome with a predominance of the central anti-cholinergic manifestations, in fact, not all the characteristics of anti-cholinergic toxidrome may be present in some cases of poisoning due to some plants having a hybrid form [3].

## Conclusion

*Atropa Belladona* acute intoxication is a severe condition with variable presentation. It's should be considered in the presence of anti-cholinergic toxidrome. Therefore, children must be informed of poisonous substances in the environment and traditional medicine should be based on scientific facts. The treatment is essentially symptomatic; Physostigmine can be used in severe intoxication.
